# Modified forelimb grip strength test detects aging-associated physiological decline in skeletal muscle function in male mice

**DOI:** 10.1038/srep42323

**Published:** 2017-02-08

**Authors:** Hikari Takeshita, Koichi Yamamoto, Satoko Nozato, Tadakatsu Inagaki, Hirotsugu Tsuchimochi, Mikiyasu Shirai, Ryohei Yamamoto, Yuki Imaizumi, Kazuhiro Hongyo, Serina Yokoyama, Masao Takeda, Ryosuke Oguro, Yoichi Takami, Norihisa Itoh, Yasushi Takeya, Ken Sugimoto, So-ichiro Fukada, Hiromi Rakugi

**Affiliations:** 1 Department of Geriatric and General Medicine, Osaka University Graduate School of Medicine, Suita, Osaka, Japan; 2 Department of Cardiac Physiology, National Cerebral and Cardiovascular Center Research Institute, Suita, Osaka, Japan; 3 Department of nephrology, Osaka University Graduate School of Medicine, Suita, Osaka, Japan; 4 Laboratory of Molecular and Cellular Physiology, Graduate School of Pharmaceutical Sciences, Osaka University, Suita, Osaka, Japan

## Abstract

The conventional forelimb grip strength test is a widely used method to assess skeletal muscle function in rodents; in this study, we modified this method to improve its variability and consistency. The modified test had lower variability among trials and days than the conventional test in young C57BL6 mice, especially by improving the variabilities in male. The modified test was more sensitive than the conventional test to detect a difference in motor function between female and male mice, or between young and old male mice. When the modified test was performed on male mice during the aging process, reduction of grip strength manifested between 18 and 24 months of age at the group level and at the individual level. The modified test was similar to the conventional test in detecting skeletal muscle dysfunction in young male dystrophic mice. Thus, the modified forelimb grip strength test, with its improved validity and reliability may be an ideal substitute for the conventional method.

Evaluation of muscle strength is an essential step for researching neuromuscular disorders by using rodent models. Multiple methods have been developed to measure muscle strength in rodents, and these methods are classified as invasive and noninvasive. The invasive methods include *in situ* and *in vitro* measurement of muscle force^1^,^2^, and the noninvasive methods include the *in vivo* measurement of muscle force^3^, wire hang test^4^, treadmill test^5^,^6^, vertical pole test^7^,^8^, swimming endurance^9^,^10^,^11^, and grip strength tests^12^,^13^,^14^. Among these methods, grip strength tests are more convenient and give less stress to animals than other tests. Thus, for more than 30 years, grip strength tests have been widely used alone or in combination with other tests to assess the phenotypes of strains of transgenic mice with neuromuscular disorders and to evaluate the effects of various chemicals on motor performance. However, as with other physical tests, grip strength tests in rodents are influenced by many factors other than pure motor function^15^. Among several types of grip strength tests, the forelimb grip strength test is a commonly used method in which an inspector horizontally pulls the tail of a rodent that grips a bar connected to a monitoring device, and the maximal value is recorded as the forelimb grip strength^13^,^14^. The factors that can interfere with correct measurement include inconsistent procedures by inspectors and the motivation of the rodent to keep gripping the bar. Specifically, the motivational factor is a major problem that may cause inconsistencies in the measurement and thus require additional experiments to be performed^7^,^15^,^16^. To address this problem, we made a simple modification to the conventional forelimb grip strength test for mice. The present study sought to clarify the reliability and validity of the modified forelimb grip strength test compared with the conventional test. Specifically, the validity of the modified test to detect a physiological decline in motor function during aging was investigated.

## Results

### The modified forelimb grip strength test produces more reliable results than the conventional test in mice

**Figure 1:**
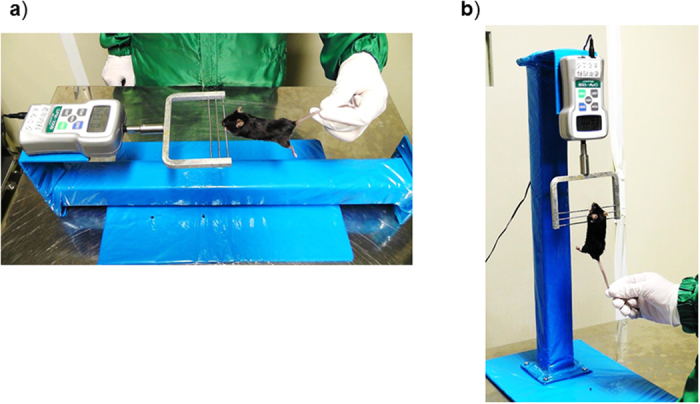
Experimental apparatuses of the forelimb grip strength tests. Experimental apparatuses of (**a**) the conventional forelimb grip strength test and (**b**) the modified forelimb grip strength test.

**Figure 2:**
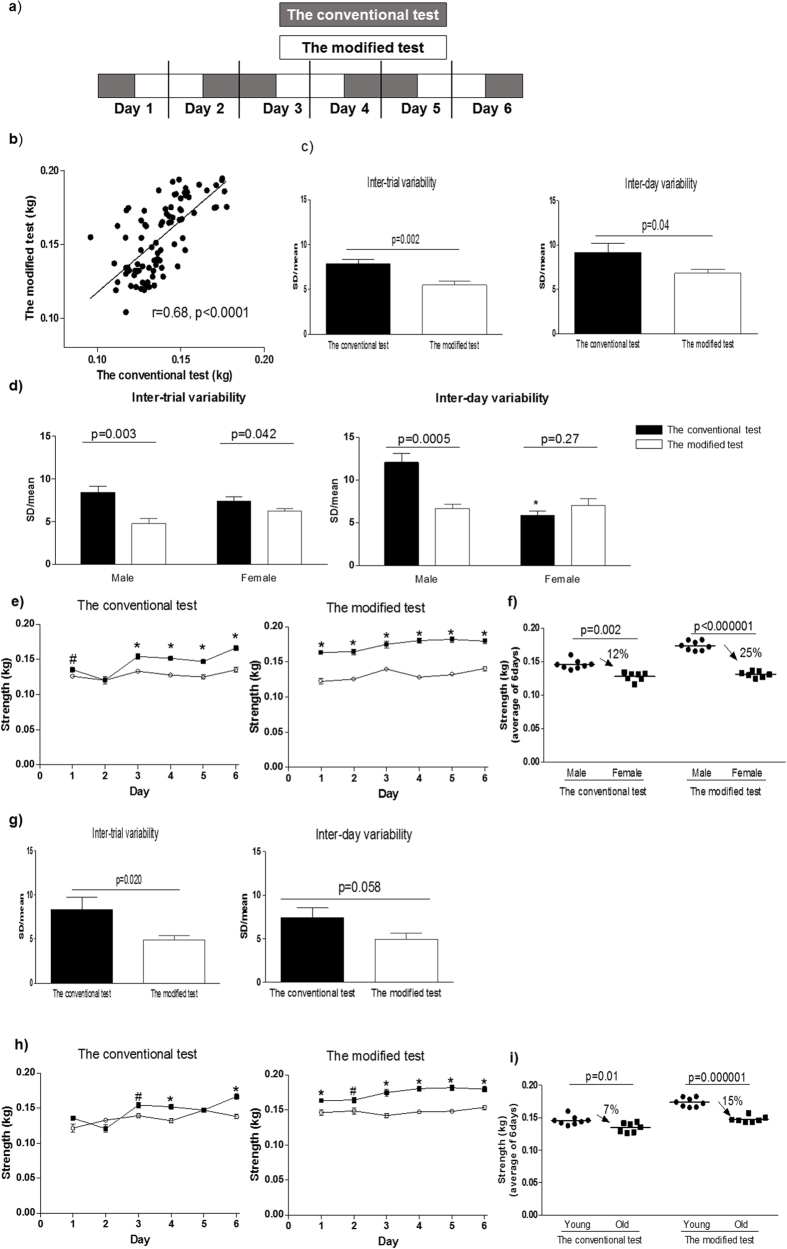
Forelimb grip strength tests over 6 days. (**a**) The sequence of two different forelimb grip strength tests. (**b**) Correlation of the average values of the measurement between the modified and conventional tests. r = Pearson correlation coefficient. (**c**) Comparison of inter-trial variability and inter-day variability between the conventional and modified forelimb grip strength tests. (**d**) Comparison of inter-trial variability and inter-day variability between the conventional and modified forelimb grip strength tests in male and female mice. *p = 0.01 vs. the conventional test in the male mice by student’s t-test. (**e**) The measurement values of the two tests in male (filled rectangle) and female (open circle) female mice. (**f**) Comparison of the average grip strength of male and female mice for 6 days using the conventional and modified tests. (**g**) Comparison of inter-trial variability and inter-day variability between the conventional and modified forelimb grip strength tests in old mice. (**h**) The measurement values of the two tests in young (16-week-old, filled rectangle) and old (2-year-old, open circle) male mice. (**i**) Comparison of the average grip strength of old and young male mice for 6 days using the conventional and modified tests. *p < 0.01 vs. the other, #p < 0.05 vs. the other Values were compared by using paired t-test (c,d,g) or student’s t-test (e,f,h,i) and are expressed as the mean ± SEM.

The new forelimb grip strength test was modified from the conventional test by rotating the system vertically. With this modification, we expected that mice would be more strongly motivated to keep grasping the bar of the equipment. The experimental apparatuses used in both tests are presented in Fig. 1a,b, and the movie of the procedures is included in the supplemental file (Supplemental video). The modified forelimb grip strength test and the conventional test were consecutively performed 6 times per day on 6 continuous days in young male (n = 8) and female (n = 7) C57BL6 mice of 16 weeks old. To prevent fatigue or habituation from influencing the experiment, the order of the two tests was alternated during the 6 days (Fig. 2a). A significant correlation in the average measurement value of each mouse was observed between the modified and conventional tests performed on the same day (Fig. 2b, r = 0.68, p < 0.01). To evaluate the reliability of the modified test, inter-trial and inter-day variability were calculated, as shown in the Methods and in Fig S1. As a result, both inter-trial and inter-day variabilities were lower in the modified test than the conventional test in these mice (Fig. 2c). When data were analyzed separately for males and females, inter-day variability of the conventional test in female mice was lower than that of male mice (p = 0.01), and the difference in inter-day variability between the two tests was not significant in female mice (Fig. 2c). These results suggest that the conventional forelimb grip strength test has better reliability in female than male mice, and the modified test improves the reliability in the conventional test especially in male mice.

### Modified forelimb grip strength test is more valid than the conventional test in detecting differences in grip strength between male and female mice

In the conventional forelimb grip strength test, there was very small or no difference in grip strength between male and female mice in the first two days, and the difference was apparent during the remaining 4 days (Fig. 2e). In contrast, the grip strengths of female mice were consistently lower than those of male mice during the 6-day period of the modified test. The average grip strengths of female mice over 6 days were significantly lower than those of male mice in both the conventional and modified tests; however, a greater difference was observed in the modified test (Fig. 2f). Our results suggest that the modified test is more valid to detect a gender-difference in motor function than the conventional test.

### Modified forelimb grip strength test is more valid than the conventional test in detecting differences in grip strength between young and old male mice

To investigate the validity of the modified test to detect aging-associated decline in skeletal muscle strength, old (2-year-old) male mice were compared to the young male mice. The inter-trial variability in the modified test was lower than the conventional test in the old mice, while the difference in the inter-day variability was not statistically significant (Fig. 2g). The inter-day variability of the conventional test in old mice was lower than that of young mice (Fig. 2d, g, p = 0.01). Figure 2h showed that the conventional test detected the difference in grip strength between young and old male mice on only 3 out of 6 days, and the results were comparable for those 3 days. In the modified test, the grip strengths of old mice were consistently lower than those of young mice during the 6-day test period. The difference of the average grip strengths over 6 days between old and young mice was greater in the modified test than the conventional test (Fig. 2i). Given that the average life span of male C57BL6 mice is approximately 26 months^17^, the reduced grip strength in old mice of 24 months old appears to reflect an age-associated reduction in motor function. Together, our results suggest that the modified test is more valid to detect an age-associated reduction in motor function than the conventional test in male mice.

### Modified forelimb grip strength test detects age-associated reduction in the motor function of individual male mice

**Figure 3:**
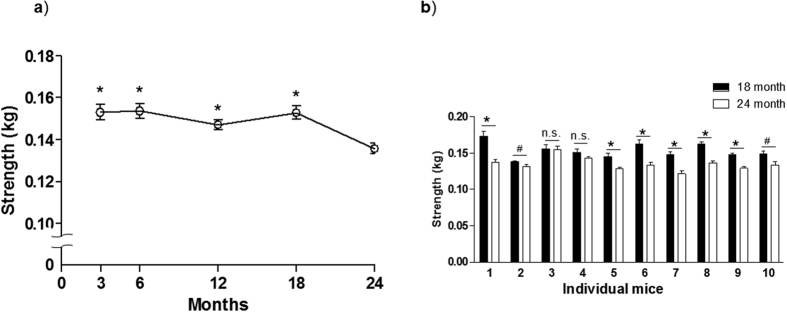
Modified forelimb grip test during the aging process in male mice. (**a**) Serial change of values measured by the modified forelimb grip strength test during the aging process in male C57BL6 mice. The same 10 mice were subjected to the modified test at 3,6, 12, 18 and 24 months of age. Measurements were performed 6 times per day for two days, and the data were averaged as an individual value. Values were compared by using one-way repeated measure ANOVA with Holm-Sidak testing. *p < 0.01 vs. 24 months old. (**b**) Comparison of grip strength in individual mice between 18 and 24 months old. The values of 1 to 10 on the horizontal axis represent mean values of 12 measurements in two days from 10 different mice. Values were compared by using Student’s t-test and are expressed as the mean ± SEM. *p < 0.01 vs. 24 months old, ^#^p < 0.05 vs. 24 months old.

**Figure 4:**
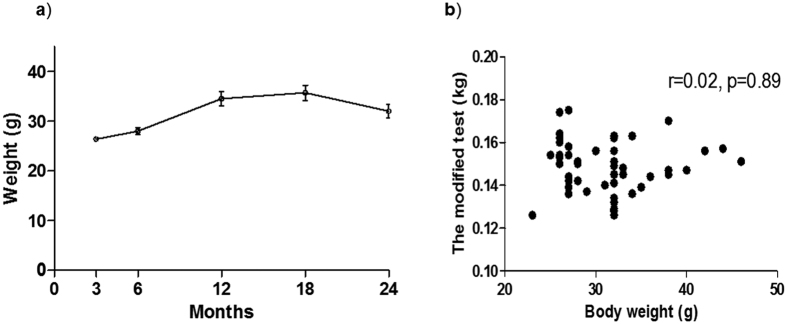
Body mass change during the aging process in mice. (**a**) Serial changes in body mass during the aging process in mice. (**b**) Correlation between values in the modified grip strength test and body mass. Values are expressed as the mean ± SEM.

To further clarify the validity of the modified forelimb grip strength test in detecting modest age-associated declines in motor function, 10 male C57BL6 mice were subjected to the modified test 3 times per day for 2 consecutive days at 3, 6, 12, 18 and 24 months after birth. The average values of the modified forelimb grip strength test were identical at 3, 6, 12 and 18 months but were significantly decreased at 24 months (Fig. 3a). When grip strengths from 6 trials at 18 and 24 months in each mouse were individually analyzed, 8 out of 10 mice showed significantly decreased grip strength at 24 months (Fig. 3b). Body weight increased until 18 months and then tended to decrease at 24 months (Fig. 4a). No significant correlation was observed between grip strength in the modified forelimb grip strength test and body mass, thus suggesting that the modified test is not affected by body weight (Fig. 4b).

### Modified forelimb grip strength test detects a decline in motor function in male dystrophic mice

**Figure 5:**
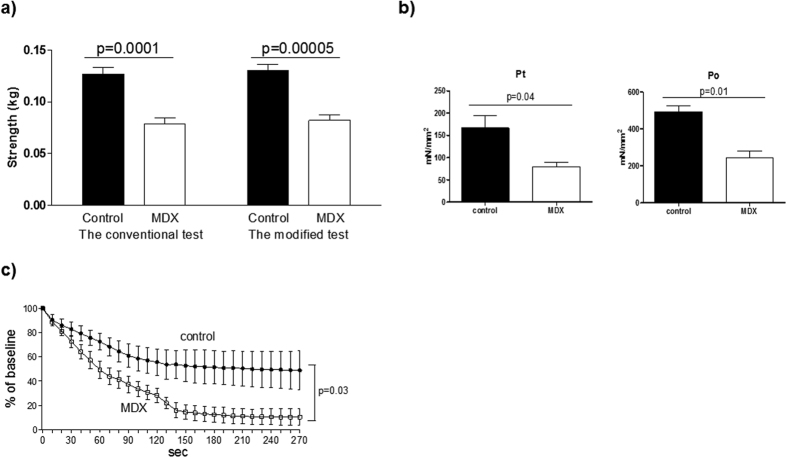
Forelimb grip strength tests in dystrophic mice. (**a**) Comparison of grip strength between seven 10-week-old male *mdx* mice with B10 backgrounds and control B10 mice by using the two different methods. (**b**) Pt (absolute twitch force) and Po (maximal isometric tetanic force) in the TA muscles of three 10-week-old male *mdx* and control mice with a B10 background. (**c**) Change in force during fatigue-inducing conditions in the TA muscles Each grip strength test was performed 6 times per day for two days, and the data were averaged as an individual value. Values were compared by using Student’s t-test and are expressed as the mean ± SEM.

Finally, the validity of the modified test was examined using animal models of Duchenne muscular dystrophy (DMD); in *mdx* mice (C57BL/10-*Dmd*^*mdx*^), the conventional grip strength test has widely been used to evaluate influences of pharmacological intervention or genetic manipulation on motor function^2^,^18^,^19^,^20^,^21^,^22^. Grip strength of seven 10-week-old male *mdx* mice with B10 backgrounds were compared with those of control B10 mice by using the conventional and modified forelimb grip strength tests. As shown in Fig. 5a, *mdx* mice showed lower grip strengths than control mice in both tests. To confirm that decreased forelimb grips strength in *mdx* mice is a reflection of systemic skeletal muscle dysfunction, mice of the same age were subjected to *in situ* tibialis anterior (TA) muscle force analysis, an established method to evaluate pure motor function; in this analysis, the sciatic nerve was electrically stimulated, and the contractile force of the TA muscle was measured. We found that the absolute twitch force and the maximal isometric tetanic force of TA muscle in *mdx* mice were lower than that of control mice, and fatigue of the TA muscle during repetitive contractions was more prominent in *mdx* mice than control mice (Fig. 5b). These findings suggest that *mdx* mice exhibit apparent hindlimb skeletal muscle dysfunction at a young age, and that decreased forelimb grip strength in these mice result from skeletal muscle dysfunction. Conversely, the modified test has an excellent validity for the detection of skeletal muscle dysfunction in dystrophic mice similar to the established conventional test.

## Discussion

Compared with the decline in neuromuscular dysfunction in transgenic mice or in rodents with injured skeletal muscle, the physiological decline of motor function with aging is a mild and gradual process. In this study, we found that the modified forelimb grip strength test improves the validity of the conventional forelimb grip strength test to detect a difference in skeletal muscle strength between male and female mice, or between young and old male mice with the excellent reliability to show reproducible results in repeated measurements. Additionally, we performed the modified forelimb grip strength test over time in normal male mice to validate the sensitivity of the modified test in detecting small changes in neuromuscular function. The modified forelimb grip strength test was able to detect only an 11% reduction in the average value between 18 and 24 months. Importantly, the modified forelimb grip strength test was able to detect a significant change in grip strength with age at both group and individual levels (Fig. 3b). Thus, the modified forelimb grip strength test may contribute to a reduction in the number of animals required for experiments to quantify motor function.

The conventional forelimb grip strength test was similarly valid to the modified test in detecting the reduction of grip strength of dystrophic mice, compared with age-matched young control mice (Fig. 5a). This finding is not surprising, because the excellent sensitivity of the conventional grip strength test to detect motor dysfunction in dystrophic mice is supported by numerous previous studies^19^,^23^,^24^,^25^. Notably we found that there was no differences in the inter-day variability between the conventional and modified tests in the young female C57BL6 mice (Fig. 2d). We found that the inter-day variability of the young female mice in the conventional test were much smaller than the male mice, suggesting that the gender difference largely affects the reliability in the conventional test (Fig. 2d). We also found that inter-day variability of the conventional test in young male mice was larger than old mice, and there was no difference in the inter-day variability between the conventional and modified test in the old mice (Fig. 2g). Together, these findings suggest that the modified test improves the reliability of the conventional test to quantify the muscle strength especially in non-elderly robust male mice. Thus, the most effective application of the modified grip strength test is the detection of physiological alterations in the motor function of non-dystrophic mice.

We found that reduced grip strength in young *mdx* mice was accompanied by impaired tetanic force of the TA muscle, which is used to determine the maximal possible muscle contraction in *in situ* muscle force analysis. Along with *in vitro* force analysis using isolated skeletal muscle fibers, the *in situ* force analysis using living animals is an established method used to assess the contractile force of individual skeletal muscles^2^,^26^,^27^,^28^. Although these invasive methods are more reliable than noninvasive tests to monitor muscle strength, in their accuracy and quantitative ability, noninvasive tests are suitable for monitoring temporal changes in muscle force in individual mice. Thus, sequential monitoring of grip strength by the modified test in combination with a final assessment of pure motor function by using invasive methods is one of the recommended options to observe the dynamics in the muscle function of living mice with high accuracy. It should also be noted that, among noninvasive tests, *in vivo* muscle force analysis reflects pure motor function than the other noninvasive tests, because the muscle force of the lower limb analyzed in the method is induced by electrical stimulation^3^. Nevertheless, grip strength tests have been more commonly used in basic studies using rodents than the *in vivo* force analysis because the *in vivo* analysis requires special equipment, skill to perform this technique, and the use of anesthesia during measurement. Thus, *in vivo* analysis is less convenient than grip strength tests. In particular, we propose that the new grip strength test is useful to monitor age-related changes in skeletal muscle function because the grip strength test can be performed safely without anesthesia, which can be a risk in old mice. It should also be noted that the forelimb grip strength test is the only established method to specifically detect forelimb skeletal muscle function, while the *in situ* and *in vivo* analysis are applicable only in lower limbs^7^. In the present study, we confirmed that reduction of motor function in MDX mice was detected both in *in situ* analysis of TA muscle and the grip strength tests, suggesting that the combination of these methods are useful in detecting systemic skeletal muscle dysfunction.

**Figure 6:**
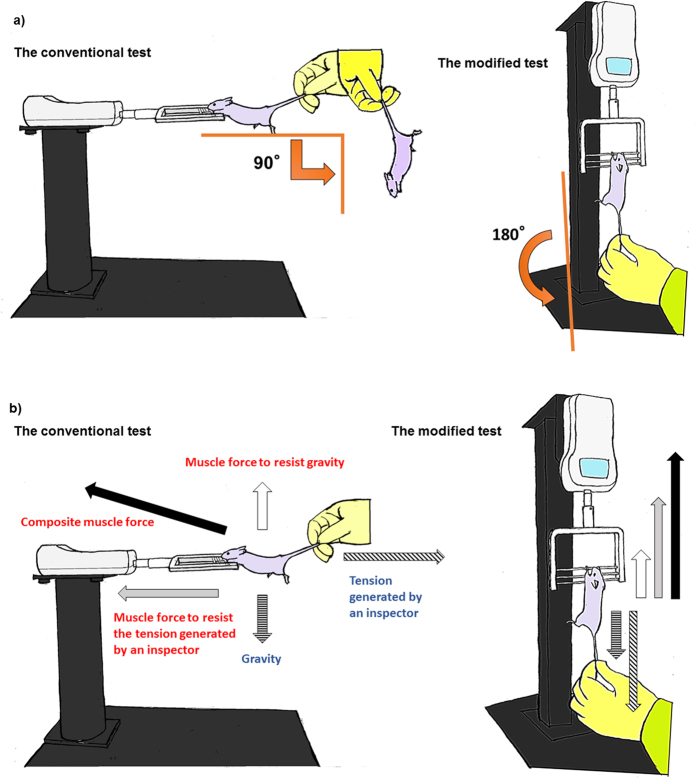
Mechanistic insights of differences between the conventional and modified methods. (**a**) Change in body angle before and after mice lost their grip. (**b**) Net force generated in the system at the time of measurement.

The only modification made in the modified forelimb grip strength test involved vertically rotating the system of the conventional test. With this modification, the tails of mice hanging onto a bar that is connected to a digital force transducer are pulled downward by an inspector, and the force applied to the bar just before the mice lose their grip is recorded as the peak tension. The reasons why this modification improves the reliability and validity of the measurements are not fully understood, but some possible explanations can be proposed. First, mice that fall from an upright position in the modified forelimb grip strength test after they lose their grip may be more fearful than mice that fall from a horizontal position in the conventional test (Fig. 6a). This difference may make mice more eager to keep gripping the bar in the modified test compared with the conventional test. In fact, we observed that male mice appeared to keep gripping the bar more insistently in the modified test than in the conventional test, and female mice appeared to grip the bar more insistently than male mice in the conventional test. Although a quantitative analysis of motivation was not performed, these observations may explain the gender difference in the reliability of the conventional grip strength test. Second, the influence of gravity on the mouse body during measurement differs between the conventional and modified forelimb grip strength tests (Fig. 6b). In the conventional test, gravity on the mouse body is directed at a right angle to the tension generated by the pull of the tail by an inspector. The competing muscle force against gravity is also directed at a right angle to the mouse muscle force required to keep gripping the bar against the pull of an inspector. Thus, the composite mouse muscle force in the conventional test is not fully conducted to the transducer, and the misconduction of the force can cause low accuracy and variability of the measured values. In contrast, in the modified test, because both the weight of the mouse body and the tension generated by an inspector are directed downward, the composite mouse muscle force in the modified forelimb grip strength test is completely conducted to the transducer, thus leading to high accuracy and low variability of the measured values. In both the conventional and modified forelimb grip strength tests, the influence of differences in mouse body mass on the measurement can be concerning. In fact, previous methodological reports have recommended calculating normalized grip strength as well as absolute strength by dividing strength by body mass^5^. However, we did not identify any association between body mass and measurement values in the modified forelimb grip strength test. In addition, we found that the changes in grip strength between 18 and 24 months in individual mice (Fig. 5b) were quite different when analyzed using strength normalized by body mass. As shown in Fig. S2, the results using normalized strength were quite inconsistent, because some mice showed significantly increased values, and others showed decreased values between periods. The normalization of grip strength by body mass is based on the assumption that body mass is proportional to muscle weight. However, the changes in body mass with aging are not necessarily proportional to the changes in muscle weight, because aging prominently changes body composition^29^. Together, our results suggest that absolute grip strength is better than body mass-normalized grip strength in detecting age-associated declines in skeletal muscle function.

We did not assess the utility of the new method in rats for this study. Our current system is not applicable to rats because the gripping bar must be fixed at a higher position for rats, because they are much larger than mice. In addition, it remains to be verified whether the measurement would be affected by variability in rat body mass. Further investigation will be required to elucidate the application of the modified forelimb grip strength test in rats.

In summary, the modified forelimb grip strength test was more reliable than the conventional test in male mice. The excellent validity of the new test is supported by the results showing that this test was able to sensitively detect a modest aging-associated decline in neuromuscular function. The low variability of the data gathered by using the new method allows for reliable results to be obtained for individual mice and enables a reduction in the number of animals required in experiments. Thus, the new method may ultimately contribute to decreasing experimental costs and to the protection of animal welfare.

## Methods

### Experimental Animals

C57BL/6 J mice were purchased from Clea Japan (Tokyo, Japan), and *mdx* mice with B10 and DBA/2 backgrounds and their respective control mice were gifts from the Central Institute of Experimental Animals (Kawasaki, Japan). Mice received standard diets, were housed in an air-conditioned room with a 12:12 light/dark cycle (light off at 8:00 PM) and were allowed *ad libitum* access to food and tap water. All experiments were approved by the Animal Care and Use Committee of Osaka University and were conducted in strict accordance with the U.S. National Institutes of Health Guide for the Care and Use of Laboratory Animals.

### Forelimb grip strength test

A Grip Strength Meter (GPM-100; Melquest, Toyama, Japan) was used to measure forelimb grip strength. As a mouse grasped the bar, the peak pull force in grams was recorded on a digital force transducer. In the conventional test, a mouse was allowed to grasp the bar mounted on the force gauge. The gauge was reset to 0 g after stabilization, and the mouse’s tail was slowly pulled back by an inspector^13^,^14^ (Fig. 1a). Tension was recorded by the gauge at the time the mouse released its forepaws from the bar. For the modification of the conventional test, the gauge was rotated vertically and fixed to the metal stand to keep the system immobilized. The measurement procedure was identical to that in the conventional test except for the direction in which the mouse’s tail was pulled by an inspector (Fig. 1b). In each test, trials in which only one forepaw, or the hindlimbs were used and in which the mouse turned during the pull or leaves the bar without resistance were excluded^30^. Given that the speed of the tail pull can influence the measurement, we conducted the procedure at a constant speed sufficiently slow to permit mice to build up a resistance against it^15^,^30^. We performed 6 consecutive measurements per day at one-minute intervals. When the conventional and modified tests were performed in the same day, mice were allowed to rest for at least 30 minutes between the two tests. The order of mice tested on each day was randomized, and the inspector was blinded to results of the previous tests. All test sessions were performed during the afternoon hours of the light cycle (11 AM to 5 PM) in the vivarium where the animals were housed. Calibration of the equipment was periodically performed by the manufacturer. The movie of two tests is attached in the supplemental file (Supplemental video).

### Evaluation of the Contractile Properties of the TA Muscle *In situ*

TA muscle contractile properties were measured by using a modification as previously described^1^,^2^. Briefly, each mouse was anesthetized and placed prone on a heated platform to maintain its body core temperature at 37 °C. The sciatic nerve was carefully dissected, tied to a suture, and cut at the proximal end. The hind limb was shaved, and the TA muscle was surgically exposed. The distal tendon was tied at the muscle tendon junction using a 2-0 suture line, and the other end of the suture was tied to the load cell (UTA-100GR, Minebea Co., Ltd. Tokyo, Japan). To fix the limb during the measurement, the patella ligament was tied to the platform with a silk suture. The contractile properties of the TA muscle were measured using the load cell connecting to a direct-coupled amplifier (SC20AZ-2, Labo support, Osaka, Japan), and the output was recorded using computer hardware (Power Lab, Nagoya, Japan). The muscle was constantly warmed up at 37 °C and kept wet during procedure. Optimal muscle length (Lo) and absolute twitch force (Pt) were determined with a resting tension between 10 and 20 g when twitch force in response to 1 Hz supramaximal stimulation to the sciatic nerve was maximal. Maximal isometric tetanic force (Po) were determined in response to supramaximal stimulation between 50 Hz and 200 Hz at Lo. Muscle fatigue was induced by repeated supramaximal stimulation once per second with 100-ms trains at a frequency of 100 Hz for 5 min^31^. The data were recorded and analyzed using the Chart software (Version 5, Ad Instrument, Nagoya, Japan). At the end of all experiments, the distal TA tendon was removed, and TA muscle weight was measured. The muscle cross sectional area was determined on the basis of a muscle density of 1.06 g/cm^3^, and a fiber length of the Lo ratio of 0.6^1^.

### Inter-trial variability and inter-day variability

The coefficients of variation (CV, standard deviation (SD)/mean) among tests and among days were calculated as inter-trial and inter-day variability, respectively (Fig. S1). Specifically, the CV of 6 trials from an individual mouse on each experimental day was calculated, and the average of 8 mice was defined as the inter-trial variability. The CV of the average measurement values during 6 experimental days in an individual mouse was calculated as the inter-day variability. Inter-trial variability during 6 experimental days and inter-day variability among 8 mice were compared between the conventional and modified tests.

### Data analyses and statistics

All data are presented as the mean ± SEM. Significant differences between two independent groups were determined by Student’s t-test. Paired t-test was used to assess differences between two variables in the same mice. One-way repeated ANOVA with Holm-Sidak testing, was used to assess temporal changes in the same mice. Pearson’s correlation coefficient was calculated to evaluate the correlations between two variables.

### Statistics for reliability and validity

Some statistical analyses were designed to clarify reliability and validity of the modified test^32^,^33^. Paired t-test was used to compare inter-trial and inter-day variability between the conventional and modified forelimb grip as a measure of reliability (Fig. 2c,d,g). Pearson’s correlation coefficient (Fig. 2b) or Student’s t-test (Fig. 5a) was used to show a criterion-validity of the modified test compared with the conventional test that has been a golden standard to assess forelimb grip strength^7^. A construct validity to reflect biological aging of skeletal muscle was shown by comparison of young and old mice by Student’s t-test (Fig. 2e,f,h,i) or by comparison of grip strength during aging in the same mice by one-way repeated ANOVA (Fig. 3a).

## Additional Information

**How to cite this article**: Takeshita, H. *et al*. Modified forelimb grip strength test detects aging-associated physiological decline in skeletal muscle function in male mice. *Sci. Rep.* **7**, 42323; doi: 10.1038/srep42323 (2017).

**Publisher's note:** Springer Nature remains neutral with regard to jurisdictional claims in published maps and institutional affiliations.

## Supplementary Material

Supplementary Information

Supplemental Video
