# Supplementary material for: Modified forelimb grip strength test detects aging-associated physiological decline in skeletal muscle function in male mice
Source: Sci Rep. 2017 Feb 8;7:42323. doi: 10.1038/srep42323 (PMC5296723; doi:10.1038/srep42323)
Supplement: Supplementary Information [file srep42323-s1.pdf]

Modified forelimb grip strength test detects aging-associated physiological decline in skeletal muscle function in male mice

Hikari Takeshita[1], Koichi Yamamoto[1]*, Satoko Nozato[1], Tadakatsu Inagaki[2], Hirotsugu Tsuchimochi[2], Mikiyasu Shirai[2], Ryohei Yamamoto[3], Yuki Imaizumi[1], Kazuhiro Hongyo[1], Serina Yokoyama[1], Masao Takeda[1], Ryosuke Oguro[1], Yoichi Takami[1], Norihisa Itoh[1], Yasushi Takeya[1], Ken Sugimoto[1], So-ichiro Fukada[4], Hiromi Rakugi[1]

1.Department of Geriatric and General Medicine, Osaka University Graduate School of Medicine, Suita, Osaka, Japan

2.Department of Cardiac Physiology, National Cerebral and Cardiovascular Center Research Institute, Suita, Osaka, Japan

3.Department of nephrology, Osaka University Graduate School of Medicine, Suita, Osaka, Japan

4.Laboratory of Molecular and Cellular Physiology, Graduate School of Pharmaceutical Sciences, Osaka University, Suita, Osaka, Japan

Supplemental video

Procedures of the conventional and the modified forelimb grip strength test are shown sequentially in the video.

Figure S1

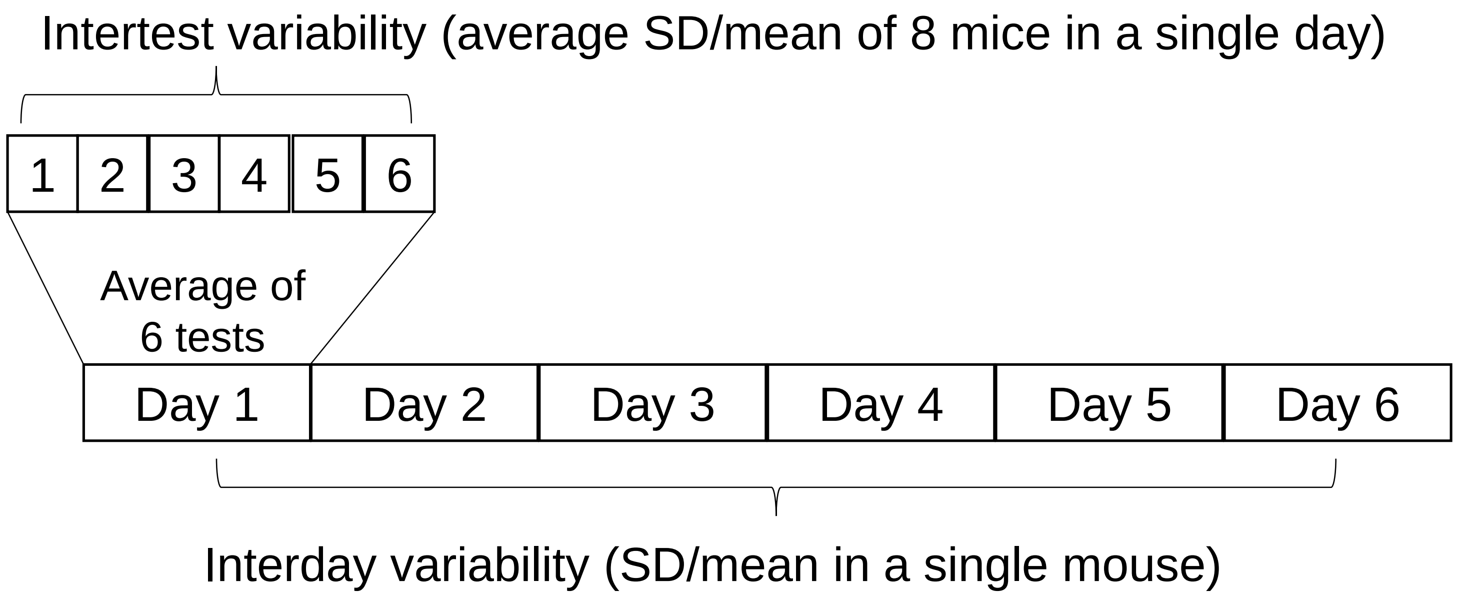

Intertest variability and interday variability

Figure S2

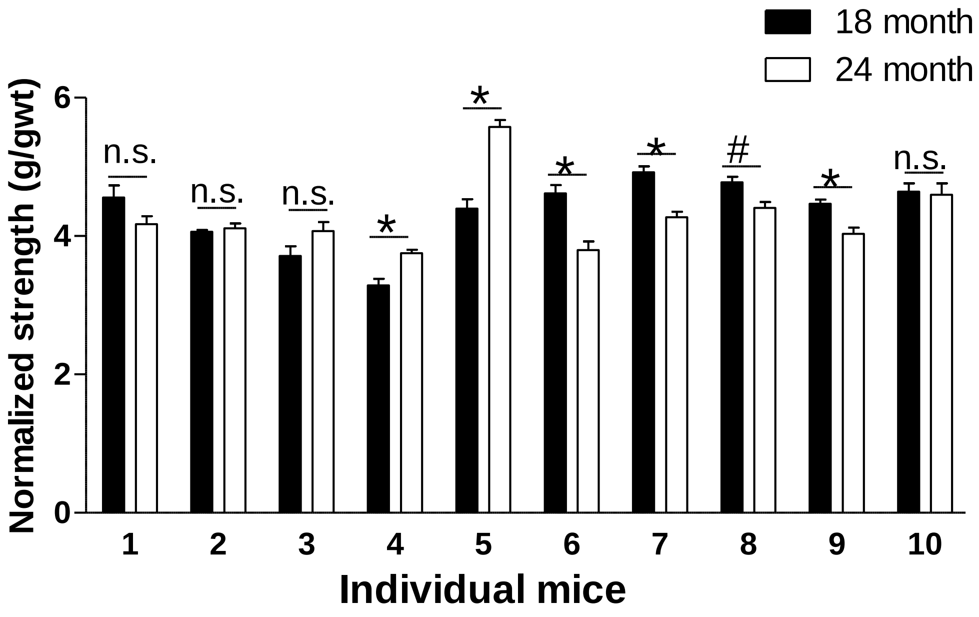

Comparison of grip strengths normalized by body mass in individual mice between 18 and 24 months. The values 1 to 10 on the horizontal axis represents values from 10 different mice.

* p<0.01 vs. 24 months old, #p<0.05 vs. 24 months old
